# T cells, adhesion molecules and modulation of apoptosis in visceral leishmaniasis glomerulonephritis

**DOI:** 10.1186/1471-2334-10-112

**Published:** 2010-05-11

**Authors:** Francisco AL Costa, Maria G Prianti, Teresa C Silva, Silvana MMS Silva, José L Guerra, Hiro Goto

**Affiliations:** 1 Departamento de Clínica e Cirurgia Veterinária, Centro de Ciências Agrárias, Universidade Federal do Piauí, Teresina, PI, Brazil; 2 Laboratório de Soroepidemiologia e Imunobiologia, Instituto de Medicina Tropical de São Paulo, Universidade de São Paulo, Av. Dr. Enéas de Carvalho Aguiar, 470, 05403-000 - São Paulo, SP, Brazil; 3 Departamento de Patologia, Faculdade de Medicina Veterinária e Zootecnia, Universidade de São Paulo, Av. Prof. Dr. Orlando Marques de Paiva, 87, São Paulo, SP, Brazil; 4 Departamento de Medicina Preventiva, Faculdade de Medicina, Universidade de São Paulo, SP, Brazil

## Abstract

**Background:**

Immune complex deposition is the accepted mechanism of pathogenesis of VL glomerulopathy however other immune elements may participate. Further in the present study, no difference was seen between immunoglobulin and C3b deposit intensity in glomeruli between infected and non-infected dogs thus T cells, adhesion molecules and parameters of proliferation and apoptosis were analysed in dogs with naturally acquired VL from an endemic area. The dog is the most important domestic reservoir of the protozoa *Leishmania (L.) chagasi *that causes visceral leishmaniasis (VL). The similarity of VL manifestation in humans and dogs renders the study of canine VL nephropathy of interest with regard to human pathology.

**Methods:**

From 55 dogs with VL and 8 control non-infected dogs from an endemic area, kidney samples were analyzed by immunohistochemistry for immunoglobulin and C3b deposits, staining for CD4^+ ^and CD8^+ ^T cells, ICAM-1, P-selectin and quantified using morphometry. Besides proliferation marker Ki-67, apoptosis markers M30 and TUNEL staining, and related cytokines TNF-α, IL-1α were searched and quantified.

**Results:**

We observed similar IgG, IgM and IgA and C3b deposit intensity in dogs with VL and non-infected control dogs. However we detected the *Leishmania *antigen in cells in glomeruli in 54, CD4^+ ^T cells in the glomeruli of 44, and CD8^+ ^T cells in 17 of a total of 55 dogs with VL. *Leishmania *antigen was absent and T cells were absent/scarse in eight non-infected control dogs. CD 4^+ ^T cells predominate in proliferative patterns of glomerulonephritis, however the presence of CD4^+ ^and CD8^+ ^T cells were not different in intensity in different patterns of glomerulonephritis. The expression of ICAM-1 and P-selectin was significantly greater in the glomeruli of infected dogs than in control dogs. In all patterns of glomerulonephritis the expression of ICAM-1 ranged from minimum to moderately severe and P-selectin from absent to severe. In the control animals the expression of these molecules ranged from absent to medium intensity. It was not observed any correlation between severity of the disease and these markers. There was a correlation between the number of *Leishmania *antigen positive cells and CD4^+ ^T cells, and between the number of CD4^+ ^T cells and CD8^+ ^T cells. In dogs presenting different histopathological patterns of glomerulonephritis, parameters of proliferation and apoptosis were studied. Ki-67, a proliferative marker, was not detected locally, but fewer apoptotic cells and lower TNF-α expression were seen in infected animals than in non-infected controls.

**Conclusion:**

Immunopathogenic mechanisms of VL glomerulonephritis are complex and data in the present study suggest no clear participation of immunoglobulin and C3b deposits in these dogs but the possible migration of CD4^+ ^T cells into the glomeruli, participation of adhesion molecules, and diminished apoptosis of cells contributing to determine the proliferative pattern of glomerulonephritis in VL.

## Background

Visceral leishmaniasis (VL) is highly prevalent throughout the world. In Brazil, it is caused by the protozoa *Leishmania (Leishmania) chagasi*, which is endemic in the Northeast and has recently spread to other regions [1].

*Leishmania *is an obligate intracellular parasite of mononuclear phagocytes. During host infection, in addition to the mononuclear phagocyte system organs the kidney is affected. Nephropathy of VL is frequent both in humans [2,3] and in dogs [4,5] presenting similar lesions, a fact that renders the study of canine VL nephropathy of interest with regard to human pathology. Until recently, studies of glomerular alterations in VL have shown the immune complex deposition as the only mechanism of lesioning [2-7]. However, studies on the pathogenesis of glomerulonephritis of other aetiologies have revealed the involvement of T cells [8-10] and adhesion molecules [8-12], and in a previous study, we detected CD4^+ ^T cells in the glomeruli in small sample of five dogs with naturally acquired VL from an endemic area [13]. Further, in a parallel study we demonstrated glomerulonephritis in 55 dogs naturally-infected with VL, characterised their glomerular alterations histopathologically, and classified into six different predominant proliferative patterns [14]. Both studies strongly suggested a participation of cell migration/proliferation, including T cells, in the pathogenesis of glomerulonephritis in VL. Nevertheless in the present study we initially addressed the possible presence of immunoglobulin and C3b deposits in glomeruli as pathogenic element but no difference was seen between these deposits in infected and non-infected dogs (see the results below) reinforcing the need to study the participation of other immune elements in the pathogenesis of glomerulonephritis in canine VL.

Cell cycle regulatory proteins have been related to the progression of glomerulonephritis [15], where Ki-67 is one such protein that is associated with cell proliferation [16,17] since it is absent in G0 phase. Since we observed predominantly proliferative patterns of glomerulonephritis, this aspect was addressed using this marker and focusing mesangial cells that may proliferate in glomeruli [17]. Alternatively, apoptosis has also been reported in the course of glomerulonephritis both in animal models and clinical kidney diseases [18], and considered essential to the recovery of the original glomerular structure determining the regression of cell numbers when a proliferative process is present [19,20]. Furthermore, several cytokines and inflammatory mediators are involved in the induction of or protection from apoptosis in the kidney[18,21,22]. Since inflammatory cells are source of many factors including TNF-α, IL-1α [22] that provide regulation of inflammatory process and induce apoptosis in cells, we have studied the expression of these molecules in glomeruli in VL dogs.

In the present study, we evaluated the participation of immunoglobulins, T cells, adhesion molecules, and proliferation and apoptosis and related cytokines TNF-α and IL-1α in the renal lesions in dogs with naturally acquired VL to better understand the immunopathogenesis of glomerulonephritis in VL.

## Methods

### Animals and diagnosis of VL

From a population of dogs presenting a positive serology for leishmaniasis during a survey by the Center for Control of Zoonosis of Teresina, Piauí, Brazil, performed from May 1996 through May 1998, 55 adult male and female dogs positive for anti-*Leishmania *antibodies were selected as previously described [14]. Briefly, the diagnosis of VL was confirmed by detecting *Leishmania *in smears of skin, spleen and popliteal lymph nodes, and/or culture of material from sternal bone marrow, spleen or popliteal lymph nodes. Eight dogs from the same endemic area without VL were used as controls. All *Leishmania*-infected dogs were routinely exterminated at the Center of Control of Zoonosis for the control of transmission of VL. The non-infected animals used as control in this study were street dogs collected to be exterminated for rabies control. Specimen sampling and euthanasia of the animals was performed under general anaesthesia using 25 mg/kg i.v. thiopental sodium (Sigma-Aldrich, USA) [23]. The kidneys were removed, renal tissues were fixed in 0.01 M, pH 7.4 phosphate-buffered 10% formalin and embedded in paraffin, and 3 μm thick sections of kidney were prepared and submitted to immunohistochemical staining and apoptosis analysis. All histological analysis was blind and done by two independent observers. The experimental protocol used in this study was approved by the Ethics Committees of all institutions involved in the study.

### Detection of CD4^+ ^and CD8^+ ^T cells, IgG, IgA, IgM and C3b, TNF-α, IL-1α, Ki-67 and M30 CytoDeath marker and adhesion molecules in renal tissue

Formalin-fixed and paraffin-embedded kidney sections were deparaffinized in xylene, rehydrated in decreasing alcohol concentrations, and incubated with 0.03% hydrogen peroxide in methanol solution for 30 minutes in the dark to block endogenous peroxidase activity. Antigen retrieval was performed using 1.2 mg/ml Tris-HCl, pH 1.0, in a microwave oven (Sanyo, Brazil) on maximum power, in consecutive cycles of 10 and 5 minutes. After washing in 0.01 M phosphate-buffered saline, pH 7.2 (PBS), the sections were treated using a Blocking Kit (Vector Laboratories, Inc., Burlingame, USA), and a protein block (Dako Corporation). The tissues were then incubated overnight at 4°C in a humid atmosphere with the different antibodies diluted in PBS: mouse, polyclonal, anti-*Leishmania amazonensis *antibody [14], diluted 1:1600 (vol:vol); mouse, monoclonal, anti-canine CD4 (VMRD, cod DH29A, Pullman, USA) and CD8 (VMRD, cod CAD46A, Pullman, USA) antibodies, diluted 1:500 (vol:vol); goat, polyclonal anti-canine IgG, IgA, IgM and C3b antibodies (10 μg/ml) (Bethyl laboratories, Montgomery, USA); mouse, monoclonal, anti-canine ICAM-1 and anti- canine P-Selectin antibodies (kindly provided by Professor C. Wayne Smith, Baylor College of Medicine, Houston, Texas, U.S.A.) (10 μg/ml); goat, polyclonal, anti-human TNF-α (10 μg/ml) (cod-sc-1347, Santa Cruz Biotecnology Corporation, California, USA); mouse, monoclonal, anti-human IL-1α (10 μg/ml) (cod-sc-9983, Santa Cruz Biotecnology Corporation, California, USA); mouse, monoclonal, anti-Ki-67 (clone MiB-1, diluted 1:75 vol:vol) (code M 7240, Dako Corporation, USA); and mouse, monoclonal, anti-M30 CytoDeath antibody diluted 1:50 (vol:vol), (cat 2140349, Roche, Mannheim, Germany). When mouse antibody was used, the reaction proceeded using catalyzed signal amplification (CSA) system-peroxidase (Dako Corporation, code K 1500, Carpinteria, USA) following protocols provided by the manufacturer. When goat and rabbit antibodies were used the reaction proceeded using streptavidine-peroxidase system (Dako Corporation, cod K 1500, Carpinteria, USA). After each incubation step, the sections were washed three times in PBS. The reaction was developed using 0.06% hydrogen peroxide and 0.3 mg/ml 3,3'-diaminobenzidine (Sigma Chemical, USA) in PBS. Counterstaining was performed using Harry's haematoxylin (Sigma Chemical, USA).

### Detection of apoptosis by terminal deoxynucleotidyl transferase (TdT)-mediated dUTP nick end labeling (TUNEL method)

A specific kit for apoptosis detection (Boehringer Manheimm, Germany) was used with the tissue sections, and the assay was performed following the protocols provided by the manufacturer. Formalin-fixed and paraffin-embedded sections were deparaffinized, hydrated and the endogenous peroxides blocked as stated above. The sections were washed in PBS, incubated sequentially with 0.1% Triton X-100 (Merck; Darmastadt, Germany) in 0.1% sodium citrate for 2 minutes on ice, with 20 μg/ml Proteinase-K in PBS for 15 min at 37°C, with 3% bovine serum albumin and 20% foetal bovine serum (Cultilab, Brazil) in PBS for 30 minutes, and then with the TUNEL mix [terminal deoxynucleotidyl transferase (TdT) and fluorescein isothiocyanate (FITC)-conjugated dUTP] in humidified chamber for 60 min at 37°C. The reaction proceeded with incubation with horse-radish peroxidase-conjugated anti-FITC antibody Fab fragment for 30 min at 37°C, and the reaction developed using 0.06% hydrogen peroxide and 0.3 mg/ml 3,3'- diaminobenzidine tetrahydrochloride (Sigma Chemical, USA) in PBS, and counterstained with Harris' hematoxylin. After each incubation step, the sections were washed three times in PBS. A negative control was performed omitting TdT in the reaction. As a positive control, the section was incubated with 1 mg/ml Deoxyribonuclease I (Gibco BRL, USA) in 50 mM Tris-HCl pH 7.5, 1 mM MgCl2, 1 mg/ml BSA for 10 minutes at room temperature.

### Morphometry

Morphometric analysis were performed on selected sections stained for distinct markers using an automatic image analyser employing Bioscan Optimas software (Optimas, Edmonds, CA, USA, Version 4.10) on a total of 50 glomeruli per animal; in a minority of samples with not enough glomeruli, cells were counted in at least 20 glomeruli. The following parameters were evaluated: cells positive for *Leishmania *antigen, CD4^+ ^and CD8^+ ^T cells, apoptotic markers M30 and TUNEL, proliferative marker Ki-67 and cells expressing TNF-α and IL-1α.

### Statistical analysis

The morphometric parameters were analysed using the Kruskal-Wallis and Dunnett's or Dunn tests to compare multiple groups, and the Mann-Whitney or Student t-test to compare two groups, employing Sigma Stat software (Jandel Corporation, USA). The semi-quantitative parameters were analysed by One-way analysis of variance and Newman-Keuls tests for the comparison of multiple groups, using GraphPad Prisma V.3 statistical software (USA).

## Results

### Detection of immunoglobulins and C_3_b in glomeruli

**Figure 1:**
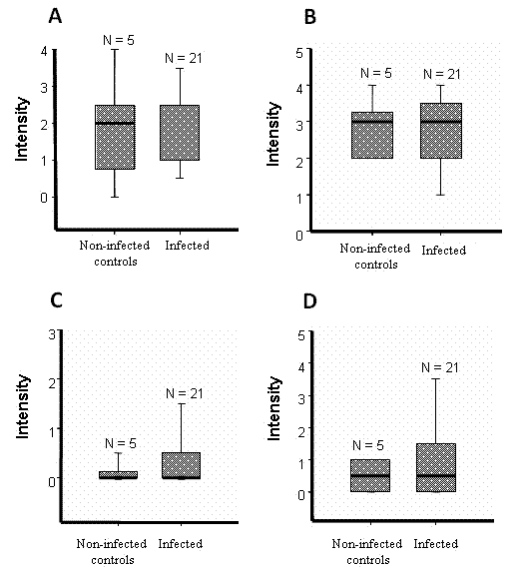
**Immunoglobulin and C3b deposits in the glomeruli of dogs with visceral leishmaniasis**. Intensity of IgG (A), IgM (B), IgA (C) and C3b (D) deposits in 21 dogs with visceral leishmaniasis and in five non-infected control dogs.

Immunoglobulins IgG, IgM and IgA and C_3_b were probed in 26 infected dogs, and in 5 non-infected, control dogs. The antigens were present in all the patterns of glomerulonephritis, and in the non-infected, control dogs. Semi-quantitative analysis of immunoglobulins and C_3_b deposits in the glomerular capillary wall showed no significant difference when a group of infected dogs was compared to the non-infected, control group (Figure 1).

### Detection of *Leishmania *antigen, CD4^+ ^and CD8^+ ^T cells in glomeruli

**Figure 2:**
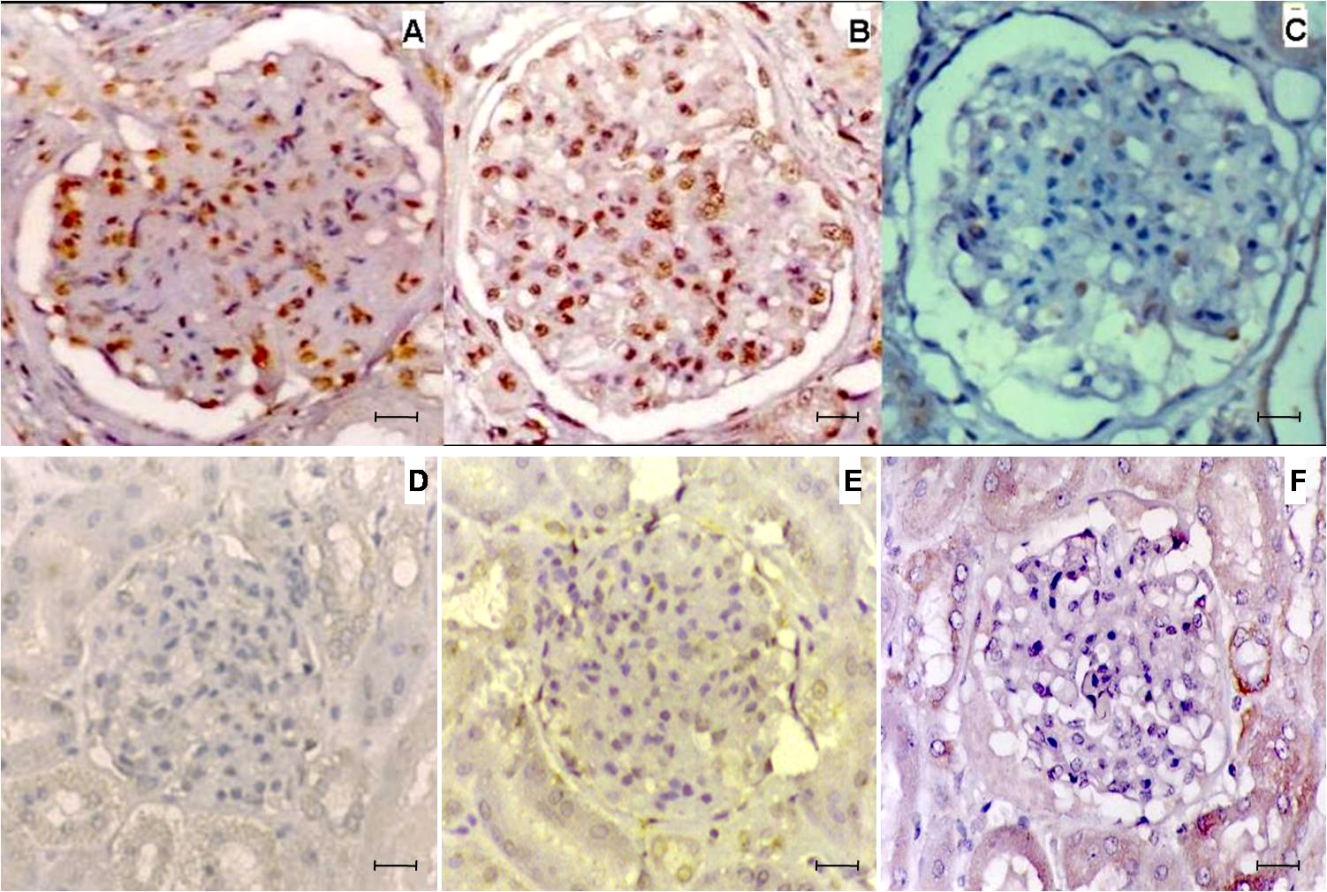
***Leishmania *antigen, CD4^+ ^T cells and CD8^+ ^T cells in the glomeruli in dogs with and without visceral leishmaniasis**. Detection of *Leishmania *antigen (A), CD4^+ ^T cells (B) and CD8^+ ^T cells (C) in glomeruli in dogs with visceral leishmaniasis. Bar = 16 μm). Absence of staining of *Leishmania *antigen (D), CD4^+ ^T cells (E) and CD8^+ ^T cells (F) in glomeruli in non-infected control dogs. bar = 25 μm. Immunohistochemistry. Different molecules when present appear stained in brown.

In dogs with VL, *Leishmania *antigen (Figure 2A) was detected in glomerular cells in 54 (98%) of 55 infected dogs. It was absent in one infected dog presenting chronic glomerulonephritis and in all eight non-infected control dogs from the same area (Figure 2D).

Of the 55 dogs with VL, CD4^+ ^T cells (Figure 2B) were observed in the glomeruli in 44 cases (80%), and CD8^+ ^T cells (Figure 2C) in 17 cases (31%), but were both absent/scarse in the non-infected control dogs (Figure 2E and 2F). In all cases exhibiting CD8^+ ^T cells, CD4^+ ^T cells were also present. CD4^+ ^and CD8^+ ^T cells were present in all patterns of glomerulonephritis except chronic glomerulonephritis.

**Figure 3:**
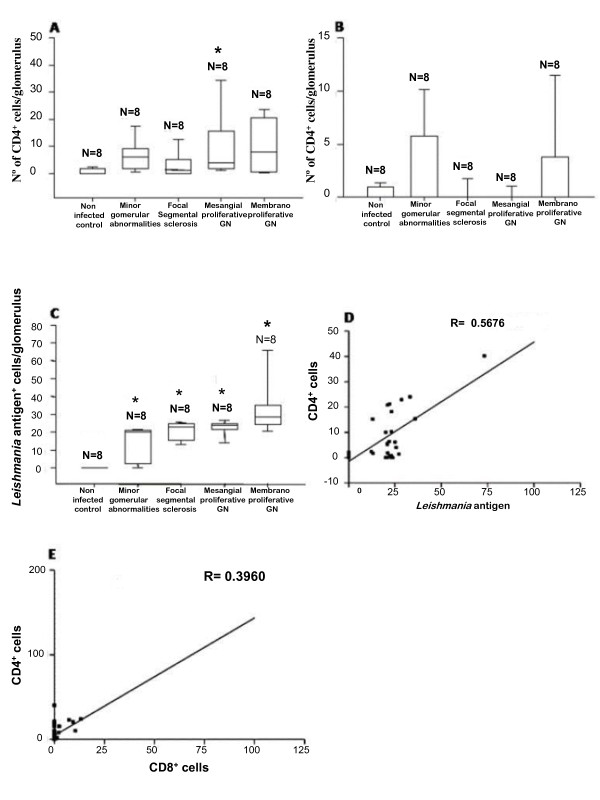
**Quantitative analysis of T cells and *Leishmania *antigen^+ ^cells and their correlation in glomeruli in dogs with visceral leishmaniasis**. (A) Number of CD4^+ ^T cells in glomeruli in VL dogs with different patterns of GN, and non-infected control animals. (B) Number of CD8^+ ^T cells in glomeruli in VL dogs with different patterns of GN, and non-infected control animals. (C) Number of *Leishmania *antigen^+ ^cells in VL infected and non-infected control dogs by glomerulonephritis pattern. (D) Correlation between the number of CD4^+ ^T cells and *Leishmania *antigen+ cells. (E) Correlation between the number of CD4^+ ^T and CD8^+ ^T cells.

CD4^+ ^T and CD8^+ ^T cells and cells stained for *Leishmania *antigen were quantified in part of samples showing different patterns of glomerulonephritis: focal segmental glomerulosclerosis (N = 8), mesangial proliferative glomerulonephritis (N = 8), membranoproliferative glomerulonephritis (N = 8), and minor glomerular abnormalities (N = 8). Significantly more T CD4^+ ^cells were observed in all infected dogs when compared with non-infected animals. However no significant differences were observed among different patterns of GN in infected dogs (Kruskal Wallis and Dunnett's tests) (Figure 3A). CD8^+ ^T cells tend to have more in infected than in non-infected dogs but the difference was not significant (Fig. 3B) Additionally, cells stained for *Leishmania *antigen were observed only in samples from infected animals independent of the pattern of glomerular alteration, even in minor glomerular abnormalities (Kruskal Wallis and Dunnett's tests) (Figure 3C). There was a positive correlation between the number of cells stained for *Leishmania *antigen and CD4^+ ^T cells (R = 0.57, p < 0.001, Spearman test) (Figure 3D), and between the number of CD4^+ ^and CD8^+ ^T cells (R = 0.40, p < 0.001, Spearman test) (Figure 3E) in the glomeruli, but no correlation was found between the cells stained for *Leishmania *antigen and CD8^+ ^T cells (data not shown).

### Detection of adhesion molecules in renal tissue

**Figure 4:**
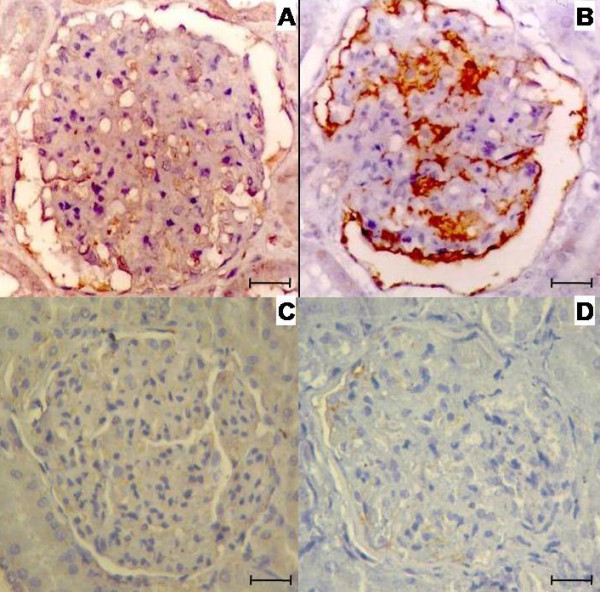
**Expression of adhesion molecules in the glomeruli in dogs with or without visceral leishmaniasis**. Expression of ICAM-1 (A) and P-selectin (B) in glomeruli in canine visceral leishmaniasis, and of ICAM-1 (C) and P-selectin (D) in glomeruli in non-infected animals. Figures A and B. Bar = 16 μm. Figures C and D. Bar = 25 μm. Immunohistochemistry. Different molecules when present appear stained in brown.

Adhesion molecules were probed in 20 infected and 5 non-infected control animals. ICAM-1 and P-selectin were present in all. In the control animals ICAM-1 was absent in only one animal and in the other control animals it was present in minimal intensity, differently what was observed in the infected animals. P-selectin was found in one control animal. Both ICAM-1 (Figure 4A and 4C) and P-selectin (Figure 4B and 4D) were localized in the endothelial lining of the glomerular capillaries, the mesangium and Bowman's capsule. In all patterns of glomerulonephritis the expression of ICAM-1 was from minimum to moderate or severe intensity and P-selectin from absent to severe intensity. In the control animals the expression of these molecules ranged from absent to medium intensity. It was not observed any correlation between severity of disease and these markers. Where ICAM-1 and P-selectin were present, primarily CD4^+ ^T cells were also detected, except in two cases of focal segmental glomerulosclerosis and in one case of membranoproliferative glomerulonephritis.

### Analysis of proliferation and apoptosis in glomeruli in dogs with visceral leishmaniasis

We analysed proliferation and apoptosis in samples from 28 dogs with VL and 7 non-infected control dogs.

Proliferative marker Ki-67 (Mib-1) antigen was detected in interstitial inflammatory infiltrate in some areas close to glomeruli but it was absent in glomerular cells both in dogs with and without VL (data not shown).

**Figure 5:**
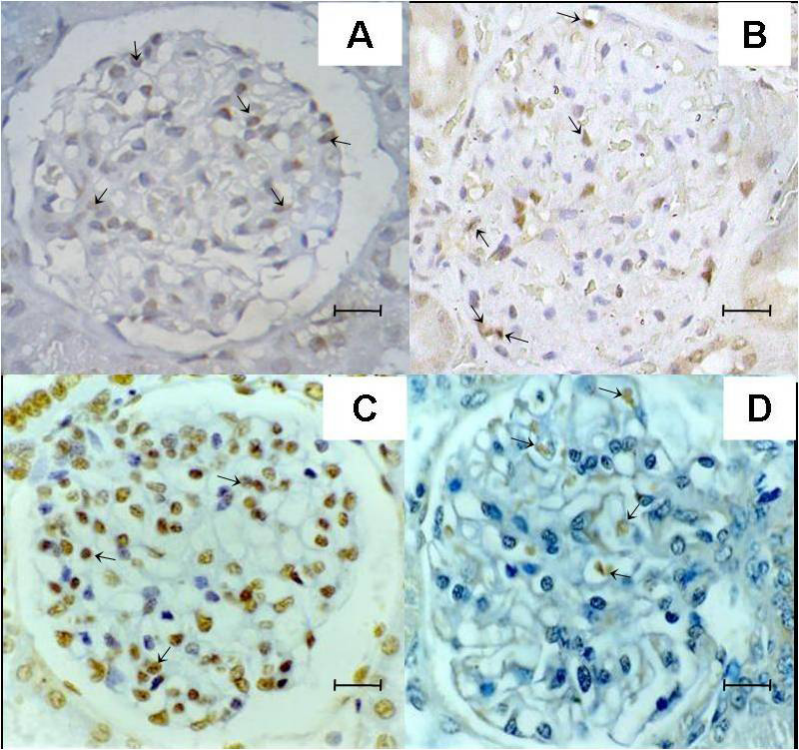
**Detection of apoptotic cells in the glomeruli in dogs with or without visceral leishmaniasis**. Detection of cytodeath marker M30 in glomerular cells in non-infected animals (A), and in dogs with visceral leishmaniasis (B). TUNEL staining in glomerular cells in non-infected animals (C), and in dogs with visceral leishmaniasis (D) Figure A. Bar = 25 μm. Figures B, C and D. Bar = 16 μm. Different molecules when present appear stained in dark brown.

**Figure 6:**
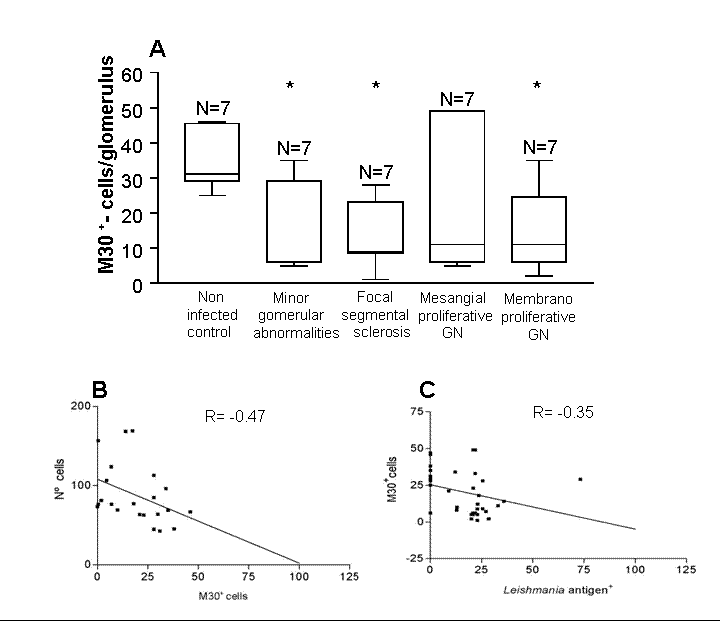
**Quantitative analysis of expression of cytodeath marker M30 and its correlation with cell number and *Leishmania *antigen^+ ^cells in the glomeruli in dogs with or without visceral leishmaniasis**. (A) Number of M30^+ ^cells in glomeruli in VL and non-infected dogs by glomerulonephritis pattern. (B) Correlation between the M30^+ ^cells and Total number of cells per glomerulus. (C) Correlation between the M30^+ ^cells and *Leishmania *antigen^+ ^cells.

Apoptosis was detected in tissue samples using two different methods: detection of the M30 cytodeath marker (Figures 5A and 5B) and the TUNEL method (Figures 5C and 5D). Apoptosis was observed in glomeruli in all 28 dogs with VL as well as in seven control animals without *Leishmania *infection. However, fewer apoptotic cells were found in infected animals than in control animals (Figures 6A, B and 6C). The results using these two methods were very similar.

Apoptosis of glomerular cells was quantified in samples submitted to detection of the M30 cytodeath marker. Significantly fewer cells stained for the M30 marker were detected in all patterns of glomerulonephritis when compared with non-infected control samples (p < 0.05, ANOVA and Newman-Keuls tests) (Figure 6A). There was a negative correlation between the number of *Leishmania *antigen^+ ^cells and the number of cells stained for the M30 cytodeath marker (R = -0.35, p < 0.001, Spearman test) (Figure 6B). There was also a negative correlation between the total number of cells per glomerulus and the number of cells stained for the M30 cytodeath marker (R = - 0.47, p < 0.001, Spearman test) (Figure 6C).

**Figure 7:**
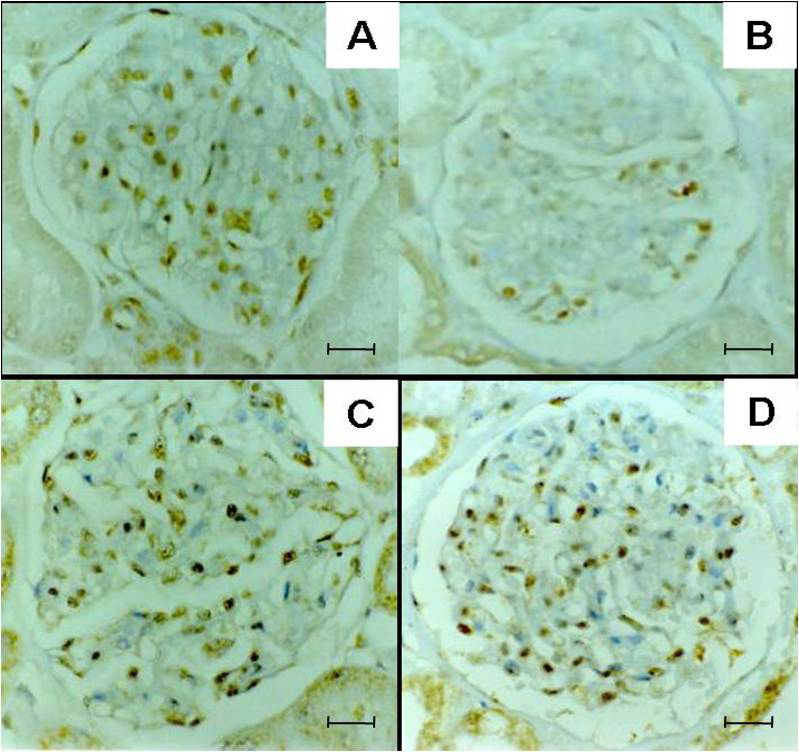
**Expression of TNF-α and IL-1 in the glomeruli in dogs with or without visceral leishmaniasis**. Expression of TNF-α in glomerular cells in non-infected dogs (A), and dogs with visceral leishmaniasis (B). Expression of IL-1α in glomerular cells in non-infected dogs (C), and in dogs with visceral leishmaniasis (D). Figures A and B. Bar = 25 μm. Figures C and D. Bar = 16 μm. Immunohistochemistry. Different molecules when present appear stained in brown.

**Figure 8:**
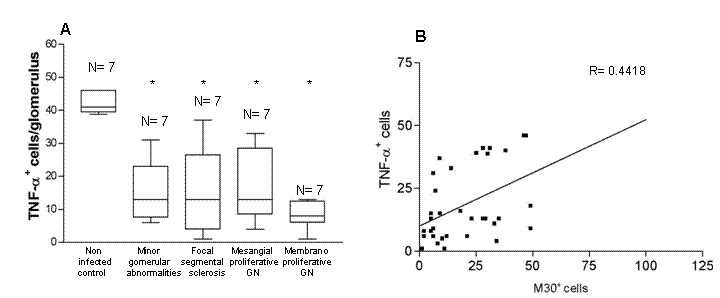
**Quantitative analysis of cells expressing TNF-α and its correlation with M30^+ ^cells**. (A) Number of cells expressing TNF-α in glomeruli in infected and in non-infected dogs. (B) Correlation between the number of cells expressing TNF-α and M30^+^cells.

Apoptosis-related cytokines were investigated and TNF-α was detected in the endothelial lining of the glomerular capillaries, in the mesangium, and on mononuclear cells in glomeruli. TNF-α was expressed in all animals studied. The expression of TNF-α in 28 animals with naturally acquired VL was less than in the seven non-infected control animals (Figures 7A and 7B). IL-1α was detected in the endothelial lining of the glomerular capillaries, mesangium and in mononuclear cells in glomerulus (Figures 7C and 7D). However the intensity was similar in the 28 VL dogs when compared with the seven non-infected control dogs. When cells expressing TNF-α were quantified, fewer cells were detected in samples of VL dogs presenting different patterns of glomerulonephritis than in the non-infected control animals (p < 0.05, (Kruskal Wallis and Dunn's tests) (Figure 8A). Furthermore, there was a positive correlation between the number of cells expressing TNF-α and M30 cytodeath marker (R = - 0.44, p < 0.001, Spearman test) (Figure 8B).

## Discussion

Since the accepted pathogenic mechanism of glomerulonephritis in visceral leishmaniasis is immune complex deposition, in the present study we probed initially for immunoglobulin, C_3_b and *Leishmania *antigens. Immunoglobulins and complement deposits were not present in greater quantities in the glomeruli of infected dogs compared to non-infected, control dogs. Canine VL is considered to undergo chronic evolution, and therefore, these findings suggested that immunoglobulin and complement play no role in the pathogenesis of glomerulonephritis in infected dogs, at least in the apparently advanced stage in which the studied animals were examined. Further, some studies in literature have shown that the immune complex is not considered important in the pathogenesis of glomerulonephritis, reinforcing our findings. Levels of immune complex detected in the bloodstream of dogs and humans with visceral leishmaniasis does not correlate with the nephropathy of VL [5,24,25]. The absence of IgG, IgA and IgM deposits in the kidney has been reported in some human VL [26]. Further, similar to our findings, immunoglobulins have also been detected in samples from control kidney in a study of human VL [2]. However, in experimental visceral leishmaniasis in the hamster, IgG deposits were found in greater intensity than in control cases in certain phases of the infection [27]. Thus we cannot completely discard such participation in the pathogenesis of canine VL during other periods of infection.

If immunocomplex deposition is not the pathogenic mechanism, other mechanisms may be operating. The finding of focal segmental glomerulosclerosis, a pattern not caused by immune complex, suggests other mechanisms of glomerular injury [28]. Further there is growing evidence that T cells and adhesion molecules play a fundamental role in the pathogenesis of certain immunologically-mediated glomerulonephritis [9,11,29-35]. Therefore, the presence of these immune elements was investigated in dogs naturally infected with VL in the present study. We found a considerable presence of CD4^+ ^T cells in the glomeruli of 44 (80%) infected dogs, but absent/scarse CD4^+ ^T in the non-infected control dogs. The presence of CD8^+ ^T cells was less noteworthy. These findings suggest a role for CD4^+ ^T cells in the pathogenesis of glomerulonephritis in canine VL, as predicted in our preliminary study [13]. Further in contrast we did not observe any significant difference in the intensity of immunoglobulin deposit in infected versus non-infected dogs.

Detection of the *Leishmania *antigen in glomeruli in 98.2% of the infected dogs strongly suggests that the glomerular lesions are caused by *Leishmania *infection. The *Leishmania *antigen was present in phagocytic cells, probably mesangial cells occupying the mesangial region. In addition, the positive correlation observed between the presence of the *Leishmania *antigen and CD4^+ ^T cells suggests that the *Leishmania *antigen may guide the inflammatory infiltrate of CD4^+ ^T cells in the glomeruli in canine VL. Furthermore, in experimental and human tegumentary leishmaniasis, data reinforce the pathogenic role of CD4^+ ^cells in lesion development of leishmaniasis [36,37].

P-selectin and ICAM-1 were detected in the most of samples in canine VL. Expression of ICAM-1 was reported in certain human glomerulonephritis and in murine malaria [31,33,38-40]. Strong expression of P-selectin in the mesangium, in the glomerular capillaries and Bowman's capsule was also found in other human and experimental glomerulonephritis [41-43], and the expression of P-selectin in the glomeruli was suggested to be critical for control of the severity and diversity of glomerular lesioning [12,44]. The detection of P-selectin in the mesangium, associated with the strong presence of CD4^+ ^T cells but absence of polymorphonuclear leukocytes in the glomeruli suggests that newly migrated platelets may be present in the glomeruli besides CD4^+ ^T cells that express P-selectin. Furthermore, an interaction between P-selectin and sub-populations of lymphocytes and platelet aggregation were seen preceding the inflammatory cell infiltration and intraglomerular cell proliferation [43,45]. The fact that CD4^+ ^T cells, adhesion molecules and *Leishmania *antigen were concomitantly present in these samples suggests their complementary role in pathogenesis.

Despite the majority of studies suggesting that the hypercellularity in glomerulonephritis is due to the increased cell proliferation [46,47], in the present study Ki-67 antigen in the renal lesions in dogs with naturally acquired VL was not significantly expressed, suggesting no important proliferative process ongoing in these cases. This result suggested that the maintenance of glomerular hypercellularity in canine VL must be due either to the inhibition of apoptosis in mesangial cells or migration of inflammatory cells or both. Since we observed mononuclear cells, mainly CD4^+ ^T cells, in glomeruli in canine VL but their absence in controls, we concluded that these cells migrated into the glomeruli in VL cases. In addition, apoptosis was examined as a mechanism by which surplus mesangial cells are cleared [18-20].

In the present study we detected apoptosis using two different methods. M30 staining detects cytokeratin 18 cleavage by caspase with generation of a neo-epitope at an earlier stage of apoptosis [48]. The second method, TUNEL, detects apoptosis when DNA fragmentation takes place at later stage. This method is also supposed to stain proliferative cells in culture, but other studies show that this rarely happens in tissue so it is thus more specific for apoptosis [49-51]. Similarity of the data based on these two methods reinforces our findings. We observed less apoptosis in glomerulonephritis in canine VL that may contribute to the persistence and progression of glomerular hypercellularity compared to other pathologies [52,53]. It was observed in different patterns of glomerulonephritis, and a negative correlation was seen between the presence of the *Leishmania *antigen and M30 staining. These data suggest a role of the parasite component in this process, similar to the protection from apoptosis of macrophages seen when infected by *Leishmania *[54].

In our control sample, the frequency of cells undergoing apoptosis was relatively high which could be due to the likely contact of control dogs with different infectious agents present in the environment.

As cells undergoing apoptosis were more frequently observed in control than in *Leishmania*-infected animals, and since T cells were absent in the glomeruli of control animals, we believe that these cells were probably mesangial cells.

There are few studies in the literature on apoptosis in trypanosomatid infections, and none on leishmaniasis. In myocarditis of experimental canine Chagas disease, abundant apoptosis of myocytes, endothelial cells, and immune effector cells including lymphocytes was observed [55]. In human chronic Chagas' heart disease, apoptosis of inflammatory cells has been observed and it is suggested to be related to the clearing of lymphomononuclear cells in the lesion [56].

Inflammatory cells are source of many factors including TNF-α, IL-1α, IFNγ, Fas ligand, oxygen radical species and nitric oxide that provide regulation of inflammatory process and induce apoptosis in cells, as observed in renal parenchymal cells and in bovine glomerular endothelial cells [57-59]. We studied TNF-α and IL-1. We detected TNF-α on mesangial cells, endothelial cells, Bowman's capsule and inflammatory infiltrate cells in glomeruli in canine VL. Our data contrast with the detection of TNF-α mRNA only on inflammatory cells in another study [60]. In dogs with VL, the TNF-α expression was lower than in non-infected control animals. Since there was a positive correlation between the expression of the TNF and M30 cytodeath marker, it may suggest induction of apoptosis through the TNF receptor in the kidney.

Although the receptors for TNF and IL-1 are different, the post-receptor events may be similar for both cytokines in some situations [58]. In the present study, expression of IL-1α was studied and it was similar in infected and in control animals showing diverse results compared with that of TNF-α.

In naturally infected dogs from endemic area for VL, we observed hypercellularity in glomeruli and presence of CD4^+ ^T cells, in addition to CD8^+ ^cells, to a lesser extent. The data showing no proliferation in glomeruli suggest that migration of the inflammatory cells takes place in conjunction with adhesion molecules. In addition, the maintenance of increased inflammatory cells in glomeruli may be partly due to the decreased apoptosis seemingly related to the low expression of TNF. Ongoing further studies on experimental models of visceral leishmaniasis may clarify the time course and interplay of different immune elements in the pathogenesis of glomerulonephritis.

## Conclusion

Data from the present study suggest that T cells, mainly those CD4^+^, play a role in the immunopathogenesis of GN in VL. Besides, diminished apoptosis may maintain the proliferative GN pattern. Further studies on experimental models of VL may clarify the time course and interplay of different immune elements in the pathogenesis of GN in VL.

## Competing interests

The authors declare that they have no competing interests.

## Authors' contributions

FALC and MGP contributed equally to this work, participated in the initial conception of the study, sample harvest, performance of the assays, data analysis and manuscript preparation. TCS and SMMSS participated in sample harvest, performance of assays and discussion of the data. JLG participated in discussion of the project, technical support and contributed to the manuscript preparation. HG conceived the study and coordinated all steps and procedures of the present study, from sample harvest, performance of different assays, and participated in analysis of data and manuscript preparation. All authors read and approved the final manuscript.

## Pre-publication history

The pre-publication history for this paper can be accessed here:

http://www.biomedcentral.com/1471-2334/10/112/prepub
